# Trends in Venture Capital Investment in AI-Driven Biopharmaceutical Startups

**DOI:** 10.2196/84968

**Published:** 2026-02-25

**Authors:** Abhishek Bazaz, Yunan Ji, Mariana P Socal, So-Yeon Kang

**Affiliations:** 1 School of Medicine Johns Hopkins University Baltimore, MD United States; 2 McDonough School of Business Georgetown University Washington, DC United States; 3 Health Policy and Management Bloomberg School of Public Health Johns Hopkins University Baltimore, MD United States; 4 Carey Business School Johns Hopkins University Baltimore, MD United States; 5 Department of Health Management and Policy School of Health Georgetown University Washington, DC United States

**Keywords:** venture capital, artificial intelligence, biotechnology, biopharmaceutical, drug discovery, discovery tools, investment trends, health care innovation, startups

## Abstract

This study analyzes 2010-2024 venture capital trends in international artificial intelligence–driven biopharmaceutical startups, revealing rapid growth in discovery tool investments and concentrated US funding in California and Massachusetts.

## Introduction

Artificial intelligence (AI) is increasingly recognized for its potential to reduce costs and improve efficiency across biopharmaceutical research and commercialization [1]. Previous studies have demonstrated increasing venture capital (VC) investment in the biopharmaceutical industry and, separately, in companies that utilize AI technology [2,3]. However, little systematic knowledge exists about which biopharmaceutical sectors attract VC investment for AI-related innovation, the magnitude of these investments, and the geographical distribution of funding. A clearer understanding of this investment landscape can inform biomedical researchers and entrepreneurs seeking funding for their AI-related ventures as well as policymakers designing financial incentives [2]. We examine recent trends in VC investment into AI-driven biopharmaceutical startups.

## Methods

We used proprietary investment data from PitchBook, a leading third-party investment data provider that tracks VC deals across countries [4,5]. From January 2010 to December 2024, we identified VC-funded biopharmaceutical deals and extracted size, date, and financing type. We classified companies by using PitchBook’s primary industry designation and supplemented this with subindustry categories (eg, drug discovery) to improve specificity. This approach has been widely adopted in prior peer-reviewed research on VC investment in health care and biopharmaceuticals [2,6,7]. All capital data were adjusted for inflation by using the Consumer Price Index and expressed in 2024 US dollars. Companies were classified as AI-related if artificial intelligence or AI were disclosed in the company’s area of strategic business focus in PitchBook and non-AI otherwise. Companies were further stratified by headquarter location and primary business area according to Pitchbook: (1) biotechnology companies (developing specific therapeutics or biologics, eg, CardioGen Sciences); (2) drug discovery (computational or experimental platforms to identify new therapeutics for in-house development, eg, Aquemia); (3) drug delivery (technology for administering drugs in a clinical setting, eg, Particle Therapeutics); and (4) discovery tools (enabling technologies, devices, or software for drug discovery by other companies, eg, Carterra) [5]. The median (IQR) was calculated for deal counts and total capital invested. The compound annual growth rate was calculated for capital investment, and chi-square analysis was conducted for company industry distribution. Analyses were conducted using RStudio (version 2023.12.0; Posit). Additional methodological details are provided in Multimedia Appendix 1.

## Results

We identified 28,269 VC deals involving biopharmaceutical companies between January 1, 2010, and December 31, 2024. Of these, 1679 (5.93%) deals were associated with companies disclosing AI as a business focus. Among AI-related deals, drug discovery was the most common business focus (639/1679, 38.06% of the deals, median investment size US $9 million, IQR 3.00-30.00 million), followed by biotechnology/pharmaceutical (625/1679, 37.22%, median US $3.51 million, IQR 1.20-14.10 million), discovery tools (401/1679, 23.88%, median US $4.64 million, IQR 1.39-15.00 million), and drug delivery (14/1679, 0.83%, median US $5.23 million, IQR 4.38-11.63 million). The industry composition of VC-funded companies differed significantly between AI-related and non-AI companies. In particular, AI-related companies had a substantially higher share in discovery tools (401/1679, 23.88% vs 879/26,590, 3.31%) and minimal involvement in drug delivery (14/1679, 0.83% vs 1093/26,590, 4.11%) compared with non-AI companies, respectively (*P*<.001, Figure S1 in Multimedia Appendix 1). The increase in the number of VC deals across both AI and non-AI sectors was most reflected in early-stage deals. In 2024, 77.42% (96/124) of AI and 68.21% (723/1060) of non-AI VC deals were early-stage. Total capital demonstrated more equal growth trends across deal stages (Figure S2 in Multimedia Appendix 1).

**Figure 1:**
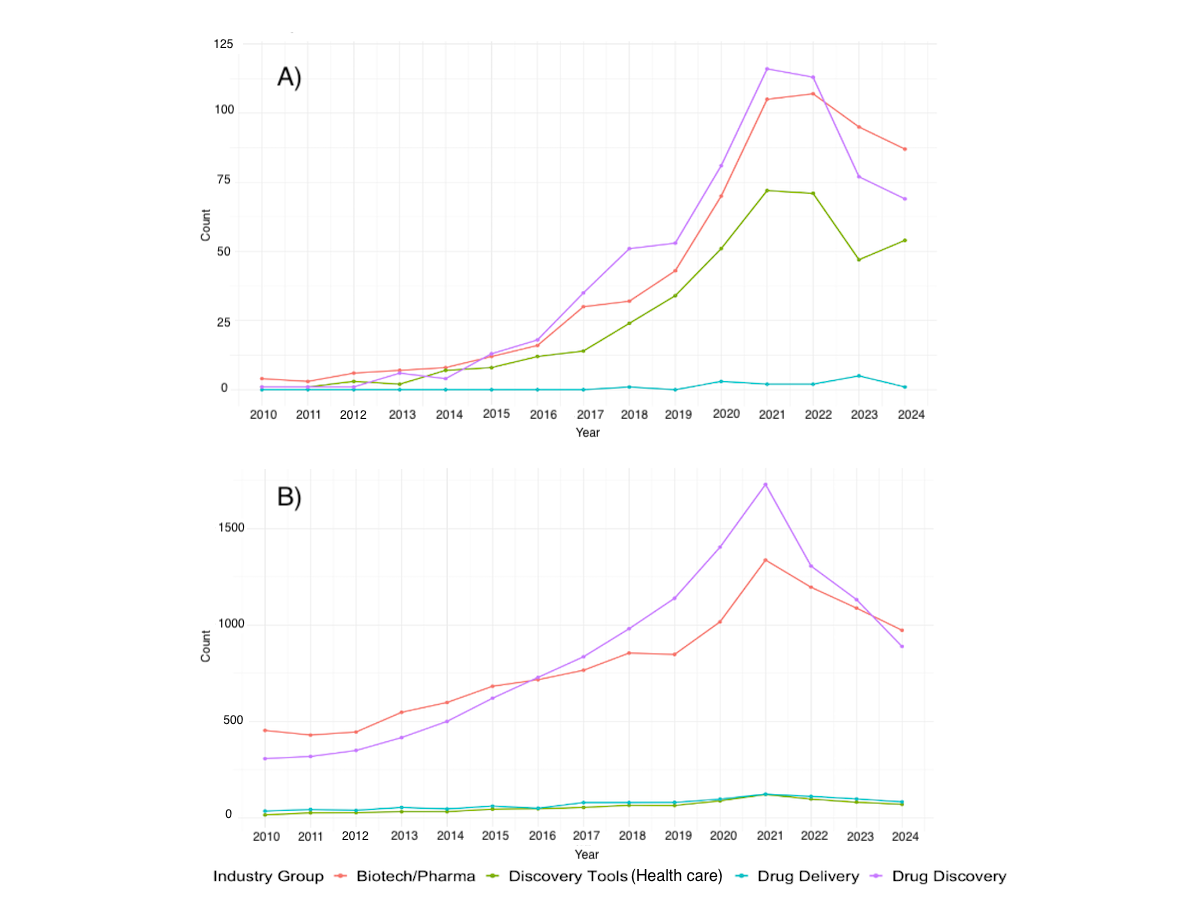
Trends from 2010 to 2024 in deal counts of venture capital deals by primary investment area: companies with artificial intelligence (AI) vs without AI. (A) AI deals and (B) non-AI deals.

Figure 1 depicts the annual deal count for biopharmaceutical startups. AI companies, both overall and within specific industries, demonstrated greater growth in VC compared with non-AI companies based on compound annual growth rate calculations (Table S1 in Multimedia Appendix 1). Among AI companies, discovery tool companies experienced the largest growth, with the median deal size of AI discovery tool companies increasing from US $0.19 million to US $7.50 million between 2010 and 2024. Their share of AI-related capital invested increased from 0.25% (0.19/76.86) to 21.85% (906.15/4147.92) from 2010 to 2024. For non-AI investments, discovery tools slightly increased from 0.87% (59.25/6816.51) to 1.58% (573.03/36,233.1) (Figure S3 in Multimedia Appendix 1).

**Figure 2:**
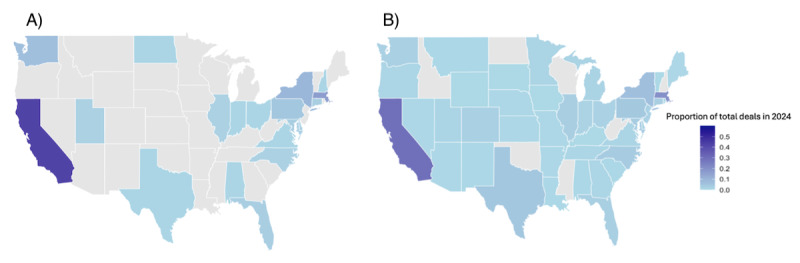
Geographic distribution of venture capital deal count involving biopharmaceutical companies in 2024. (A) AI deals and (B) non-AI deals. AI: artificial intelligence.

Figure 2 illustrates the geographic distribution of VC deals in the United States in 2024. Approximately 50.74% (852/1679) of AI-related and 46.07% (12,249/26,590) of non-AI–related deals were made for US-based companies. Nearly 60% (65/110) of US-based AI deals in 2024 were in California and Massachusetts, while non-AI companies spanned 42 states. Median AI-related deal sizes varied regionally—US $10.76 million (IQR 4-39.85 million) in California compared to US $2.94 million (IQR 1.00-14.70 million) in Connecticut.

## Discussion

While AI-related biopharmaceutical companies represent a minority of overall deals, they have experienced substantial growth, especially in discovery tools, where funding surged nearly 36-fold from 2010 to 2024. The majority of this growth occurred during and after the COVID-19 pandemic, in which VC funding experienced significant volatility [8]. AI-related VC activity expanded to 19 states by 2024, though investments remained highly concentrated in California and Massachusetts. These trends suggest that AI is an emerging investment focus but may be influenced by regional innovation ecosystems and biopharmaceutical research resources. Policymakers and state governments seeking to attract VC investment should consider emulating the frameworks used in these states, including research and development incentives and efficient regulatory pathways for AI-driven biotechnology ventures [9]. Future research evaluating specific regulatory and innovation policies that influence the diffusion of AI-driven health care companies and investment flows as well as the impact of AI on such innovation warrants consideration. Limitations of this study include its reliance on proprietary data based on publicly disclosed deals and self-reported business profiles. Companies that do not publicly report their financing activities or strategic focus may be missing from our dataset. Additionally, firms that do not explicitly identify AI as a core business area may be excluded, while some companies that use the term as a marketing tool (“AI-washing”) are included [10]. However, our classification based on companies’ strategically disclosed business focus on AI is more conservative than methods that include companies using AI only in their internal operations when estimating VC flows into AI-driven companies.

## Appendix

Multimedia Appendix 1 Details of the methodology and results.

## Abbreviations

AI: artificial intelligence
VC: venture capital

## Data Availability

The datasets generated or analyzed during this study are not publicly available due to their proprietary nature and restrictions under a data use agreement. However, the analyses can be reproduced by researchers with access to the same subscription-based data sources.
