# Supplementary material for: Trends in Venture Capital Investment in AI-Driven Biopharmaceutical Startups
Source: J Med Internet Res. 2026 Feb 25;28:e84968. doi: 10.2196/84968 (PMC12980058; doi:10.2196/84968)

**Methods**

**Data Search Criteria**

Our analysis uses the proprietary PitchBook, which provides flat text data under a data-use agreement. Our classification relied on the “verticals” field, which identifies a company’s stated area(s) of strategic business focus as disclosed to PitchBook and company website. As noted, companies were classified as AI-related if “Artificial Intelligence”, “artificial intelligence” or “AI” appeared in this field; all others were classified as non-AI. Considering the widespread use of PitchBook among VC investors and entrepreneurs, we expect companies using AI as a strategic focus to indicate it publicly. The classification was fully automated and did not involve manual review.

Our primary unit of observation was a single VC financing round. We restricted our dataset to VC deals only and used the unique Deal ID in the PitchBook Academic Data Feed to identify distinct transactions. Because syndicated rounds and co-investments involving multiple investors are recorded under a single Deal ID, restricting analyses to unique IDs prevents double counting of individual financing events. Each round was assigned to a temporal period using the Deal Date variable, and total capital invested was quantified using the Deal Size field. Consistent with our objective of capturing sustained VC activity, follow-on rounds and repeat investments were treated as distinct observations, as each represents a renewed capital commitment. To focus exclusively on equity-based VC financing, we excluded venture debt, private investments in public equity (PIPEs), secondary transactions, and unconverted convertible notes.

**Appendix Results**

**Figure S1. Chi-squared test comparing industry composition between AI and non-AI companies**

|  | Biotech/Pharma | Discovery tools | Drug delivery | Drug discovery |
| --- | --- | --- | --- | --- |
| AI | 625 (37.2%) | 401 (23.9%) | 14 (0.8%) | 639 (38.1%) |
| Non-AI | 11957 (45.0%) | 879 (3.3%) | 1093 (4.1%) | 12661 (47.6%) |

χ^2^ = 1572.1, p <0.001

**Figure S2. Trends from 2010 to 2024 in deal count and total invested capital for AI- and non-AI-related companies, stratified by funding round. (A)**

**Number of AI deals, (B) number of non-AI deals, (C) total capital for AI deals, (D)**

**total capital for non-AI deals.**

**
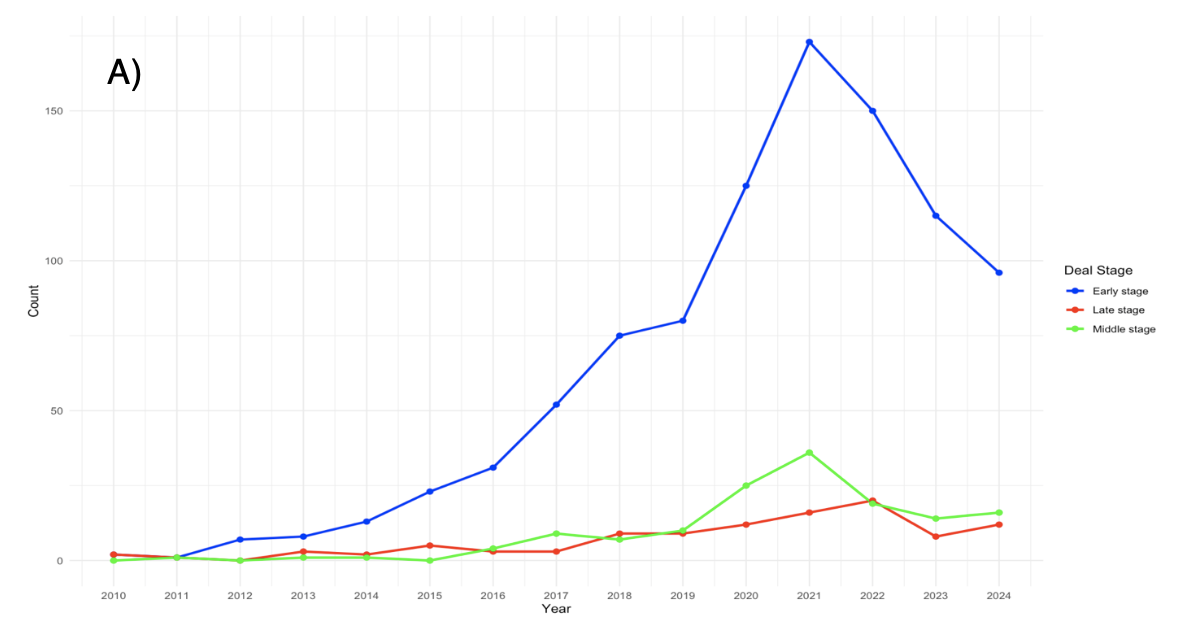
**


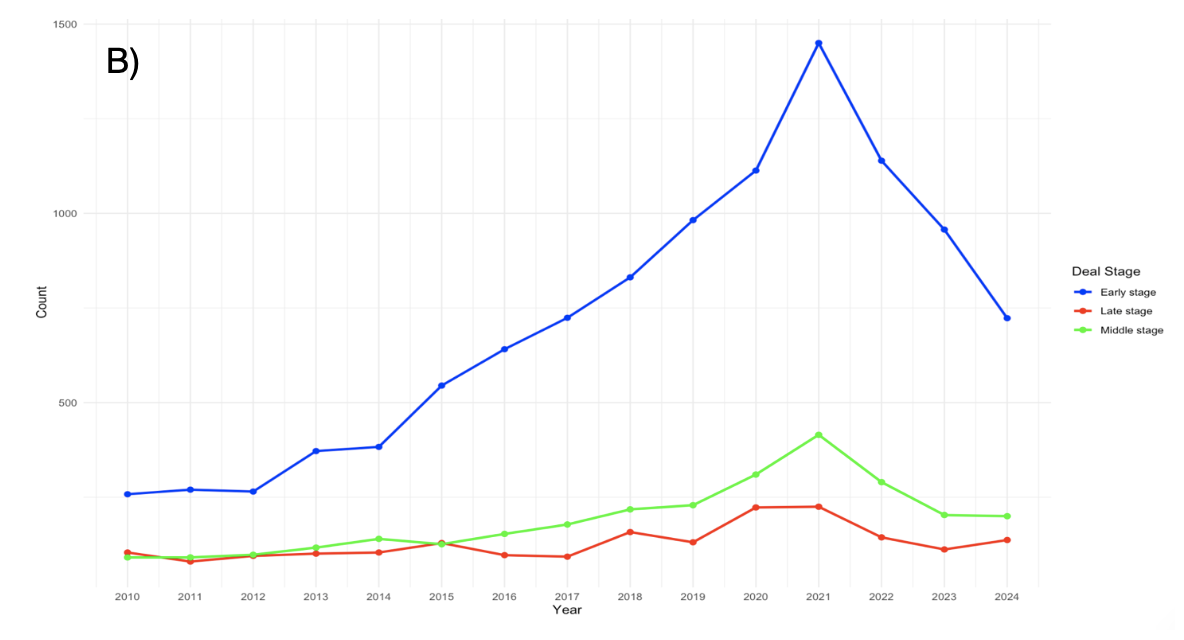


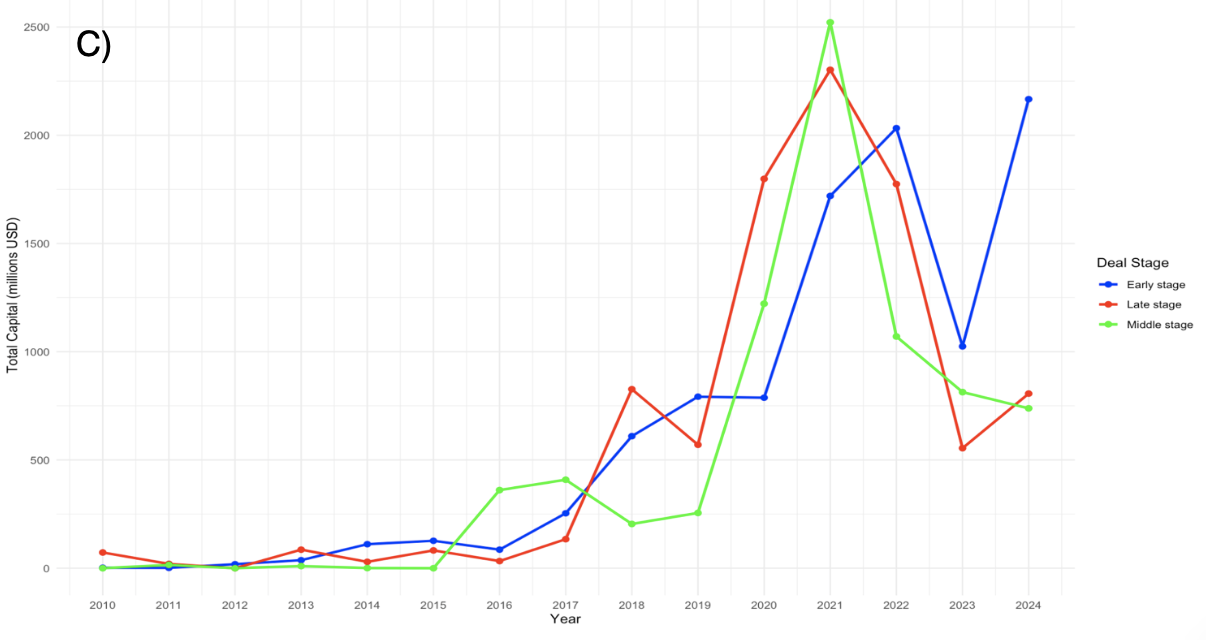


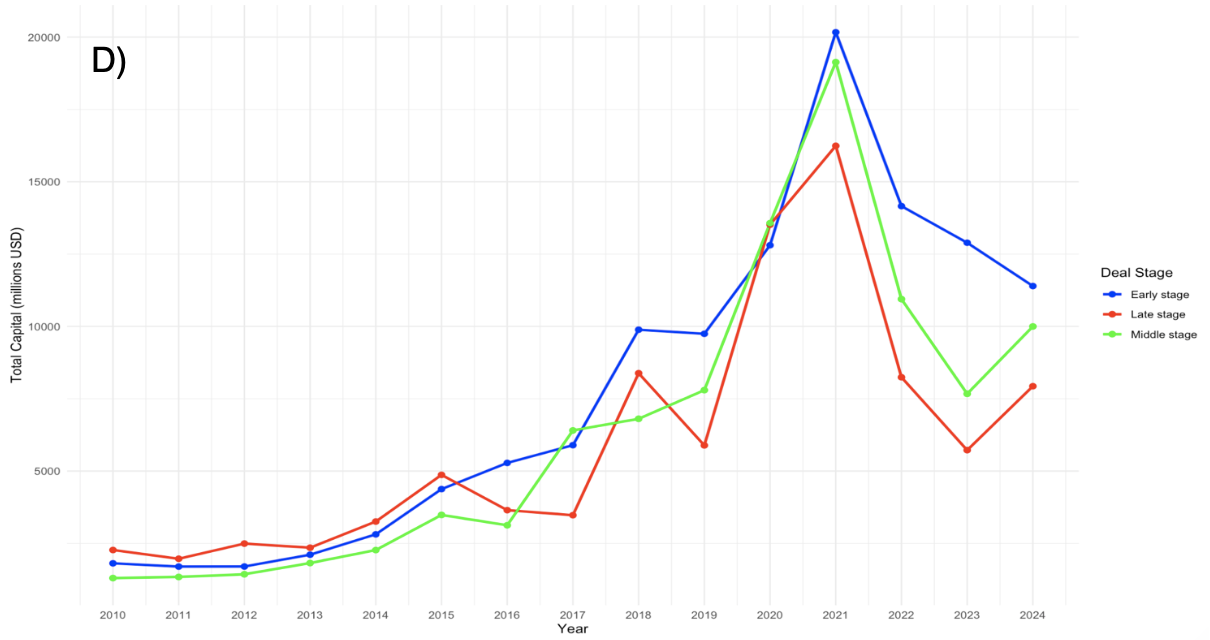


**Table S1. Compound Annual Growth Rates (CAGRs) for total capital invested in AI-related versus non-AI-related companies (2015-2024), total and stratified by industry**

|  | AI-related investment (%) | Non-AI-related investment (%) |
| --- | --- | --- |
| Overall | 34.2 | 7.91 |
| Drug discovery | 50.3 | 11.63 |
| Biotech/Pharma | 19.3 | 3.19 |
| Discovery tools | 34.1 | 13.3 |
| Drug delivery | 0 | -2.00 |

**Figure S3. Total capital invested in AI- and non-AI-related companies in 2024 USD, stratified by industry. (A) AI deals and (B) non-AI deals.**


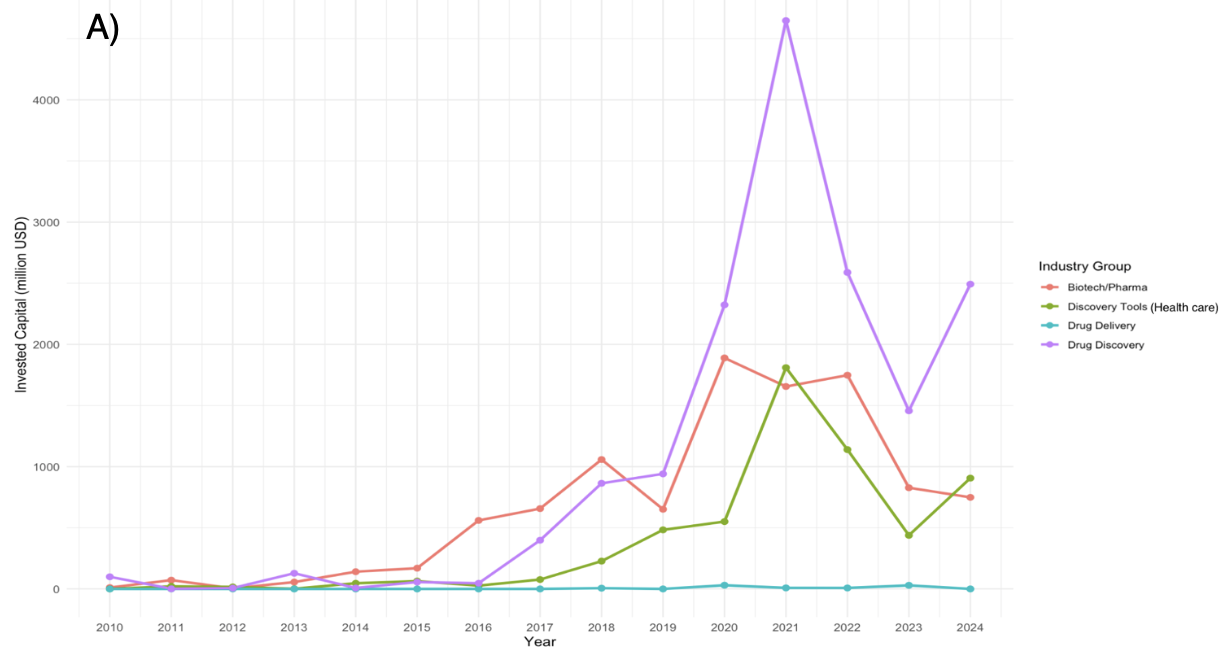


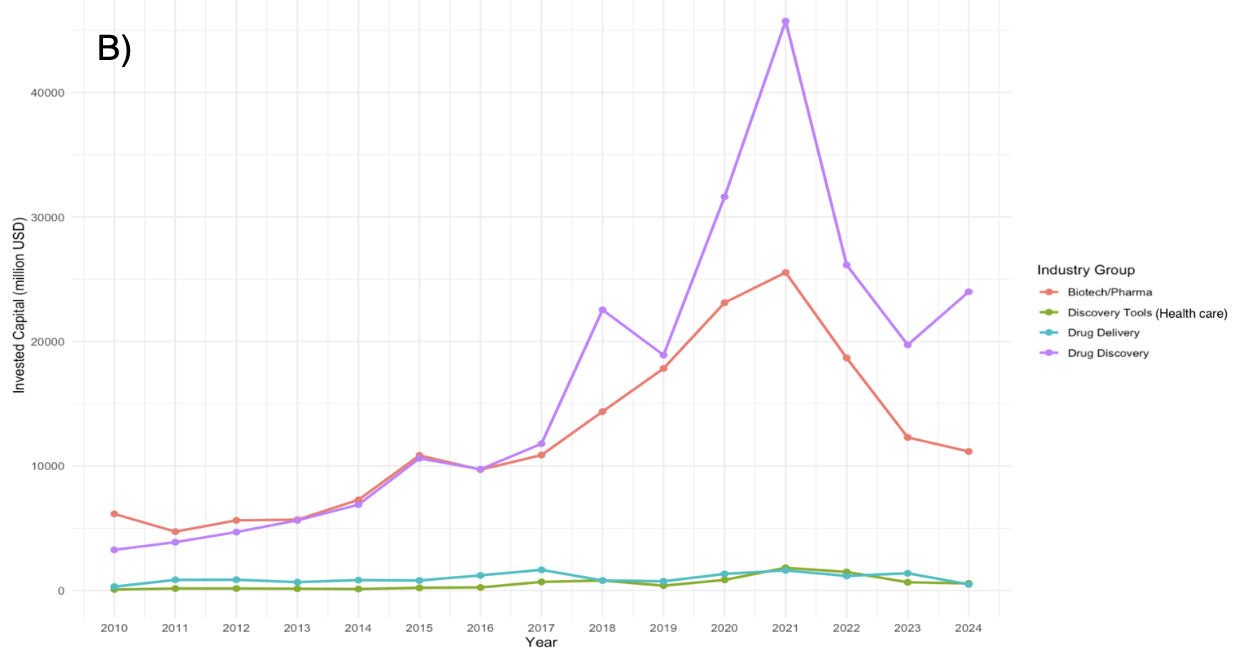

Supplement: Multimedia Appendix 1 [file jmir_v28i1e84968_app1.docx]
